# Re: A prototype non-invasive urodynamic test to estimate voiding reserve in normal adult males. By Shafik Shoukry, Mostafa Elmissiry, Ahmed Abulfotooh, Ahmed Moussa, Wally Mahfouz, Waleed Dawood, Aly Abdel-Karim and Mohamed Hassouna

**DOI:** 10.1080/2090598X.2019.1642619

**Published:** 2019-08-29

**Authors:** Hassan Shaker

**Affiliations:** Urology, Ain Shams University, Cairo, Egypt, hassanshaker@live.com

The idea of non-invasive urodynamics has been alluring to researchers and clinicians for a very long time for obvious reasons. The concept of detrusor muscle reserve appeared in the literature but without clear consensus on its definition and normal values. The authors have done a good job at trying to address this point. They took this concept one step further, trying to quantify it in a non-invasive way. As the authors mention ‘This initial prototype test is still very crude and needs further improvement’ [1]. It only serves as a proof of concept on which further research and refinements can be based. By no means, can this work can be taken as it stands to clinical practice. Furthermore, results cannot be used for comparison or as a reference except in a similar setup.
